# Amylase-Sensitive Polymeric Nanoparticles Based on Dextran Sulfate and Doxorubicin with Anticoagulant Activity

**DOI:** 10.3390/polym11050921

**Published:** 2019-05-25

**Authors:** Nikolay A. Pyataev, Pavel S. Petrov, Olga V. Minaeva, Mikhail N. Zharkov, Oleg A. Kulikov, Axeksandr V. Kokorev, Ekaterina P. Brodovskaya, Ivan A. Yurlov, Ilya V. Syusin, Andrey V. Zaborovskiy, Larisa A. Balykova

**Affiliations:** 1 National Research Ogarev Mordovia State University, 68 Bolshevistskaya Str., Saransk 430005, Republic of Mordovia, Russia; petrovps83@gmail.com (P.S.P.); polinanew@mail.ru (O.V.M.); mikhail.zharkov.92@mail.ru (M.N.Z.); oleg-kulikov-84@mail.ru (O.A.K.); kitten-777@mail.ru (E.P.B.); ivanuyrlov@mail.ru (I.A.Y.); ilya.sysin@gmail.com (I.V.S.); larisabalykova@yandex.ru (L.A.B.); 2 National Research Nuclear University MEPhI (IATE MEPHI), 31 Kashirskoe shosse, Moscow 115409, Russia; kav2972@yandex.ru; 3 Moscow State University of Medicine and Dentistry named after A.I. Evdokimov, 20/1 Delegatskaya Str., Moscow 127473, Russia; azabor@mail.ru

**Keywords:** doxorubicin, dextran sulfate, nanoparticles, amylase, cytotoxicity, ascitic hepatoma, heparin-like effect

## Abstract

This study looked into the synthesis and study of Dextrane Sulfate–Doxorubicin Nanoparticles (DS–Dox NP) that are sensitive to amylase and show anticoagulant properties. The particles were obtained by the method of solvent replacement. They had a size of 305 ± 58 nm, with a mass ratio of DS:Dox = 3.3:1. On heating to 37 °C, the release of Dox from the particles was equal to 24.2% of the drug contained. In the presence of amylase, this ratio had increased to 42.1%. The study of the biological activity of the particles included an assessment of the cytotoxicity and the effect on hemostasis and antitumor activity. In a study of cytotoxicity on the L929 cell culture, it was found that the synthesized particles had less toxicity, compared to free doxorubicin. However, in the presence of amylase, their cytotoxicity was higher than the traditional forms of the drug. In a study of the effect of DS–Dox NP on hemostasis, it was found that the particles had a heparin-like anticoagulant effect. Antitumor activity was studied on the model of ascitic Zaidel hepatoma in rats. The frequency of complete cure in animals treated with the DS–Dox nanoparticles was higher, compared to animals receiving the traditional form of the drug.

## 1. Introduction

The development of functional nanoparticles with antitumor activity is one of the main trends of modern oncopharmacology. The creation of supramolecular nanostructures, with an active drug, allows us to realize the targeted delivery of substances, to a specified area. Furthermore, nanocarriers can change the pharmacokinetics and pharmacodynamics of drugs, increasing its effectiveness and reducing its toxicity. The main requirements for matrices of drug-carrier complexes are biocompatibility and biodegradability. From this point of view, dextran and its derivatives are of great interest. Despite its fairly simple structure, this polymer has found wide applications in biomedicine. The earliest use of dextran has been as a plasma replacement in hypovolemia [1,2]. In addition, dextran has been used as an antiplatelet, in violation of arterial patency [3,4].

With the development of nanopharmaceuticals, dextran and its derivatives have become widely used in the synthesis of nanoscale drug delivery systems. The reasons for the popularity of dextran are its specific physico-chemical and biological properties. Dextran is fully biocompatible and biodegradable, and also less immunogenic than protein. Therefore, it can prevent opsonization and rapid absorption by the immune cells of protein compounds, such as insulin, thrombolytic enzymes, etc. In combination with Dextran, these compounds circulate longer in the bloodstream and retain their structure and functional properties [5,6,7]. Alhareth et al. showed that Dextran could be an alternative to polyethylene glycol, as a means of reducing the immunogenicity of doxorubicin nanoparticles [8].

Dextran itself is relatively inert, but its various derivatives and copolymers have significant functional properties. Derivatives of Dextran, with different polar groups, can be used for creating complexes based on electrostatic interactions. The most widely used anionic derivatives of Dextran are dextran sulfate (DS) and dextran phosphate (DP). These can form complexes with cationic polymers—chitosan, polyarginine, polyallylamines, etc. Such complexes often have a micelle configuration and can be loaded with drugs. Micelles of DS–chitosan, containing curcumin [9], iron oxide [10], and doxorubicin [11] have been previously described elsewhere.

Anionic dextran derivative—dextran-sulfate—is widely used, in combination with various polycations, in the Layer-By-Layer technology. This technology allows us to obtain spherical microcapsules of micron and submicron size, as well as larger structures of various configurations (e.g., polymeric chambers). Both microcapsules and chambers can be loaded with various biologically active or diagnostic agents, the delivery and release of which can be controlled by various external stimuli [12,13,14,15,16,17,18,19,20].

The ionic properties of DS can be used to synthesize various compositions, not only with cationic polymers, but also directly with substances having basic properties [21]. One such substance is doxorubicin (Dox). In the work of Yousefpour and co-authors, it was shown that a simple mixing of DS and Dox led to the formation of a specific nanocomplex [22]. Xiaoyun and co-authors have obtained hydrogel from DS and Dox by adding Ca ions, and have studied the impact of these ions on the properties of the resulting product [23].

As a rule, the anticancer activity of the nanostructured form of Dox and other cytostatic drugs is implemented due to the enhanced permeability and retention (EPR) effect. This effect, first described in 1986, has been widely discussed in the literature [24,25]. However, it should be noted that the EPR effect is not realized in all tumors, and is much less pronounced in their metastases. This is the reason for the absence of the expected effect of nanostructured drugs, in clinics. In this regard, more attention is paid to the functional properties of the particles, which, one way or another, can increase the selectivity of drug delivery to the tumor. It is more common to use different targeting ligands that selectively bind to tumor cell receptors. External physical stimuli concentrating or releasing the drug at a given location, can also be applied [26,27,28].

Additionally, an interesting direction for the implementation of the effect of targeting might be the use of specific features of the anatomy, the metabolism, and the exocrine activity of tumors. From this point of view, particles based on DS–Dox have very interesting biological properties. First, they can undergo hydrolysis by amylase. Second, they might have anticoagulant properties. Both of these potential properties can be of service in chemotherapy. Amylase-sensitive drugs might be used for treatment of amylase-producing tumors.

Malignant tumors rarely produce biologically active substances, but there are some tumors which actively synthesize enzymes. In particular, some lines of ovarian cancer, lung cancer, and myeloma, which actively secrete amylase, have been described [29]. Synthesis of amylase by tumors, is associated with the expression of the *AMY2A* gene [30]. Since the secretion of amylase is not an absolute property of the above-mentioned tumors and has no special clinical significance, there have not been many specific studies to determine the frequency of this phenomenon, and its real frequency is unknown. One study [31] has found that amylase was overproduced in 39% women with ovarian cancer, and in 93.5% of patients with an increase in this enzyme, the levels of cancer were very high. Yanagitani and co-authors [32] suggested the use of amylase levels as a diagnostic marker for small cell lung cancer and ovarian cancer.

The anticoagulant effect of DS has been described for a long time [33], but has not been used in antitumor therapy. However, this effect might be useful in intracavitary chemotherapy of serous cavity tumors. These tumors, such as primary or metastatic carcinoma of the pleura, are quite common and cause fatal complications, reducing the duration and quality of life. Methods such as the indwelling pleural catheters (IPCs) have greatly expanded the treatment of recurrent malignant pleural effusion [34]. This method was implemented by installing a catheter into the pleural cavity, and continuously introducing the chemotherapeutic agent through it, for a long time. The main problem with the use of intracavitary chemotherapy was the formation of adhesions in the pleural cavity. The frequency of complete or partial pleurodesis reached 40%. Fibrin deposition caused the formation of compartments in the pleural cavity. This complicated the access of the chemotherapy drug to the exudative fluid and reduced the efficiency of evacuation. Formation of adhesion required surgery or thrombolytic therapy, which themselves carried certain risks. The creation of a chemotherapeutic drug that could prevent the formation of fibrin clots and adhesions, would increase the effectiveness of IPC [35,36,37,38].

We have not found any articles devoted to the study of the biological activity of DS–Dox particles on multicellular organisms. The purpose of this work was the development of DS–Dox nanoparticles, with characteristics that provide the possibility of use in clinics; study of their biological properties, such as amylase sensitivity and the anticoagulant effect, as well as evaluation of antitumor activity in serous tumors—ascitic Zaydel hepatoma.

## 2. Materials and Methods

### 2.1. Materials

The following reagents were used—dextran sulfate sodium (DS, Mr ~ 40000, CAS Number 42867-5G), doxorubicin hydrochloride (Dox, > 98%, CAS Number 25316-40-9), dimethyl sulfoxide (DMSO, CAS Number 67-68-5), sodium chloride (CAS Number 7647-14-5), α-Amylase (α-Amylase from human pancreas, lyophilized powder, ≥ 100 units/mg protein, CAS Number 9000-90-2), Dulbecco’s growth medium in the needle modification (DMEM), trypsin (CAS Number 9002-07-7), phosphate buffered saline (PBS, CAS Number P4417-50TAB) methylthiazolyldiphenyl-tetrazolium bromide (MTT, CAS Number 298-93-1), Acridine Orange (AO, CAS Number 10127-02-3), Propidium iodide (PI, CAS Number 25535-16-4)—Sigma-Aldrich, St. Louis, MO, USA; cellulose membrane Q1210-55 F3—Orange Scientific, Braine-l’Alleud, Belgium; fetal bovine serum (FBS, CAS Number S1820-500)—BioWest, Nuaillé, France; Versene solution—Biolot, St. Petersburg, Russia.

### 2.2. Synthesis and Purification of DS–Dox NP

Dextran sulfate samples (5, 10, 20, 30, and 40 mg) were dissolved in 2 mL of 0.15 M NaCl solution. The doxorubicin solution in DMSO (4 mL with a concentration of 2.5 mg/ml) was quickly added to the obtained solution, at room temperature, and vigorously stirred for 10 min. The resulting mixture of dextran sulfate–doxorubicin nanoparticles (DS–Dox NP) was purified by dialysis at ambient temperatures, with a pore of 12–14 kDa. Eluent: 0.15 M NaCl, ratio NP/eluent equaled to 1/50 (*w*/*w*), the duration of the procedure equaled to 24 h. For each concentration, three synthesis series were performed.

### 2.3. Characterization

#### 2.3.1. Analysis of Loading Efficiency (LE)

Loading efficiency was determined by the formula:

ω = (*m*_0_ − *m*)/*m*_0_·100% (1)

where *m*_0_ is the initial amount of Dox, *m* is the amount of non-included Dox.

The amount of non-included drug was calculated by its concentration in dialysate. It was determined by spectrofluorimetry, using a RF-5301PC Shimadsu spectrofluorometer (Kyoto, Japan), according to a previously constructed calibration graph. The excitation wavelength was 480 nm, emission wavelength was 545 nm (Supplementary data, Figure S1).

#### 2.3.2. The Morphological Characteristics of Nanoparticles

Size distribution of DS–Dox NP were determined by dynamic light scattering (DLS) on a NANO-flex device (Microtrac Inc., Krefeld, Germany). Hydrodynamic diameter and polydispersity index was calculated using cumulant analysis methods.

Measurement of the zeta potential was performed on Stabinoanalyzer (Microtrac Inc., Krefeld, Germany).

The size and morphology of the DS–Dox NP particles were studied using scanning electron microscopy (SEM) TESCAN MIRA (Brno, Czech Republic, EU).

### 2.4. In vitro Dox Release Kinetics

Dox released from the DS–Dox NP was assessed by determining the concentration of Dox in the eluent, during dialysis, through a cellulose membrane. Three series of experiments were performed. In the 1st series (control), dialysis was carried out at room temperatures, in the 2nd and 3rd—at a temperature of 37 °C, and in the 3rd series, amylase was added to the DS–Dox NP to the final concentration of 400 U/L. The reason for dose selection is given in Section 2.5.1. Dialysis was performed for 72 h, with a 0.15 M NaCl solution, at a volume ratio of DS–Dox NP/eluent equaled to 1:50. Aliquots of dialysate were taken through 0, 0.5, 1, 2, 4, 8, 10, 24, 36, 48, and 72 h. After the collection of samples, an equal amount of 0.15 M NaCl solution was introduced into the system. The concentration of released Dox was determined by the spectrofluorimetry mentioned above.

### 2.5. Cytotoxicity Study

To assess the cytotoxicity of the synthesized nanoparticles, an MTT test, and microscopic examination were performed.

#### 2.5.1. Cell Culture and MTT-Assay

Mouse L929 fibroblast cell culture was used for the cytotoxicity study. Cells were cultivated in vials, in a DMEM medium, with the addition of 10% FBS under the following conditions—5% CO_2_ atmosphere, *t* = 37 °C, and 5% humidity (incubator Sanyo MCO-170M, Japan). For the experiment, cells in the exponential growth phase were dispersed in a 96-well plate (2 × 10^3^ cells/well) in 100 μL of DMEM medium, with 10% FBS. Cells were incubated for 24 h, then the medium was replaced with the fresh one, and the test substances were added. Free doxorubicin (series 1), DS–Dox NP (series 2), and DS–Dox NP with the addition of alpha-amylase (series 3) were then compared. The concentration of Dox had decreased from 185 to 1.4 μg/mg, with 1:2 steps (185, 92.5, 46.3, 23.1, 11.6, 5.8, 2.9, and 1.4 μg/ml). In series 2 and 3, the concentrations of Dox were the same and the concentrations of DS in the DS–Dox NP was equal 610.5, 305.3, 152.6, 76.3, 38.2, 19.1, 9.5, and 4.8 μg/ml. The concentration of amylase in series 3 was equal 400 U/L. This dose was equal to 10% of amylase IC_50_, which was determined in the preliminary cytotoxicity experiment (Supplementary data, Figure S2). There were two control series. Phosphate buffer was added to the cells in the first series, and the amylase solution to those in the second. The exposure time was 24 h. After the incubation time, the control medium was replaced with the fresh one and with MTT solution, at a concentration of 5 mg/ml. The cells were incubated for 4 h and then the medium was removed, 100 μL DMSO was added, and the microplates were shaken for 20 min, until the cell monolayer completely dissolved. Optical density was measured on a microplate ELISA reader EFOS 9305 (Moscow, Russia), at a wavelength of 492 nm. Cell viability was determined as the ratio of optical density (OD) of the sample, to the control OD, expressed as a percentage.

#### 2.5.2. Microscopic Examination

For microscopic examination (Axio Zeiss Imager A1 microscope, Carl Zeiss, Jena, Germany), the cells were placed in a 6-well plate (1 × 10^4^ cells/well), in 1 mL of medium. One hour after the cells monolayer formulation, the test substances were added to the cells—doxorubicin (185 μg/mL), DS–Dox NP (DS/Dox 610.5/185 μg/mL), and DS–Dox NP (DS/Dox 610.5/185 μg/mL) with amylase (400 U/L). In the control series, amylase and phosphate buffer were added. The exposure time was 12 h. After this time, the medium was removed, the wells were carefully washed and the cells were stained with a mixture of acridine orange and propidium iodide, according to protocol [39]. The monolayer was examined in the optical microscopy mode, and in the fluorescence mode. Differentiation of the viable, necrotic, and apoptotic cells (Supplementary data, Figure S3) was performed by counting at least 200 cells. The results were expressed in percentages.

### 2.6. Study of the Effect on Blood Coagulation

The effect of DS–Dox NP nanoparticles on the coagulation system was assessed by the Activated Partial Thromboplastin Time (APTT) and Protrombin Time (PT). Studies were performed on an analyzer-coagulometer Amelung KC4 (TRINITY Biotech, Bray, Ireland). Blood samples were collected from healthy donors, stabilized with sodium citrate and centrifuged at 3000 g, to obtain plasma. Plasma was collected in Eppendorf tubes and the investigated objects (doxorubicin solution, DS, and DS–Dox NP) were added to it, in a volume ratio of 1:10. The final obtained concentration was equal to 0.005 mg/mL for Dox, 0.017 mg/mL for DS, and 0.017/0.005 Ds/Dox for DS–Dox NP. The 0.15 M sodium chloride was used as a control. The mixture was incubated for 5 min at 37 °C, then the chronometric test was performed according to the coagulometer protocol, using standard kits. Each substance was tested three times on different plasma samples.

### 2.7. Animal Experiments

All experiments on animals were approved by the local ethical committee at the National Research Ogarev Mordovia State University. The effectiveness of the synthesized substance was studied in animals with transplanted ascitic Zaidel hepatoma. The tumor strain was obtained from a collection of the Scientific Research Institute of Cytology of the Russian Academy of Sciences. In total, 60 Wistar rats weighing 180–220 g were included in the experiment.

The tumor was transplanted by intraperitoneal injection of 5 × 10^9^ tumor cells. Animals were treated by single intraperitoneal administration of the studied drugs, on the second day, after tumor transplantation. Drugs (DS–Dox NP and free Dox) were introduced at a dose of 4 mg/kg. Two series of experiments were performed. In the first series, life expectancy and the cure rate of animals were studied. In the second one, the effect of the drugs on the morphological parameters of ascites, as well as the concentration of chemotherapy drug in ascitic fluid was studied. In each of the series, animals were divided into three groups—one experimental and two control groups (10 animals in each). In the experimental group, the animals were treated with DS–Dox NP at a dose of 4 mg/kg, diluted in 2 mL of 0.15 M sodium chloride solution. Dox in the same dose and volume was introduced to animals of the first control group and 2 mL of isotonic chloride solution sodium was used in the second control group.

#### 2.7.1. Evaluation of the Antitumor Effect

To evaluate the antitumor effect, the animals were observed for 30 days, the number of live and dead animals was recorded daily. The antitumor effect was evaluated by the following indicators: (1) animal survival—the percentage of animals that survived to 30 days; (2) average life expectancy (for dead animals); and (3) increase in life expectancy (ILE). ILE was calculated by the formula:

ILE = (LEE-LEC)/LEC·100%(2)

where LEE is life expectancies in the experimental group and LEC is life expectancies in the 2nd control group.

#### 2.7.2. Assessment of Ascitic Fluid

To estimate the volume and morphology of ascitic fluid, animals were euthanized two days after the administration of the chemotherapy agent (four days after tumor inoculation). Ascitic fluid was obtained by dissecting the euthanized animals. The volume of the fluid and its protein content were determined using the standard kit (AGAT, Moscow, Russia). The presence and weight of fibrin clots were also evaluated. The cells were counted with the counting chamber. The number of tumor cells, leukocytes, and erythrocytes was counted separately. The study of the concentration of Dox in ascitic fluid was performed by the spectrofluorimetry described above.

### 2.8. Statistics

The IC_50_ was calculated using the GraphPad Prizm 7.00 software. The significance of differences between parameters was assessed using the Wilcoxon W criterion and the Cruskell–Walysis criterion (Analysis of variance-ANOVA) using Statistica 10.0. Differences were considered significant when the *p* value was below 0.05. The results have been presented as mean ± SD.

## 3. Results and Discussions

### 3.1. Сharacteristics of DS–Dox NP

**Figure 1:**
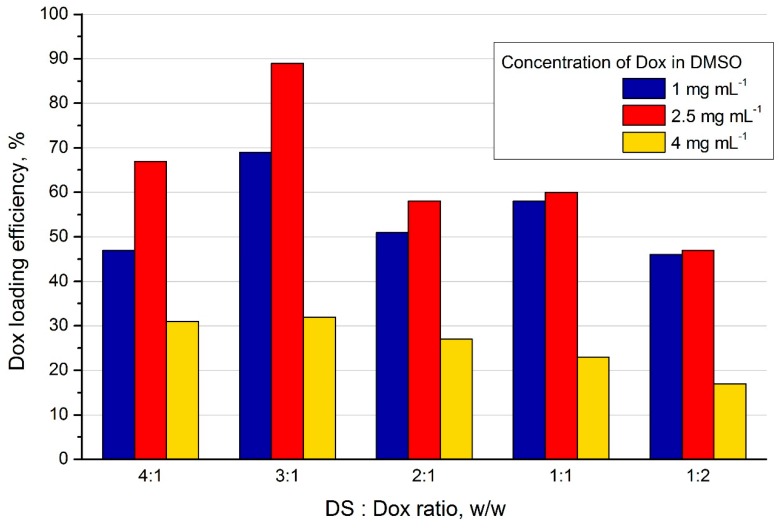
The dependence of Dox loading efficiency on the mass ratio DS/Dox.

A necessary condition for a suitable drug composition for in vivo use, is the sufficient content of medicine in a single unit dosage form. In a study by Yousefpour et al. [22], a method for the synthesis of particles of DS–Dox NP, in the aqueous phase, was described. The method was simple; however, the drug concentrations in the resulting product were more than one order of magnitude less than those needed to create clinically applicable therapeutic systems (about 2 mg/mL in solutions for intravenous administration [40]). We have attempted to reproduce this method using large concentrations of reagents. It was not possible to obtain stable systems (Supplementary data, Table S1). In this regard, we used a different method of synthesis, based on “solvent replacement”. The variable parameters of synthesis were the concentration of the polymer in the aqueous solution and the concentration of Dox in DMSO. The characteristics of DS–Dox NP obtained under various synthesis conditions are shown in Table 1 and Table S2 of the supplementary data. Figure 1 shows the dependence of the Dox loading efficiency on the mass ratio and the reagents concentration.

**Figure 2:**
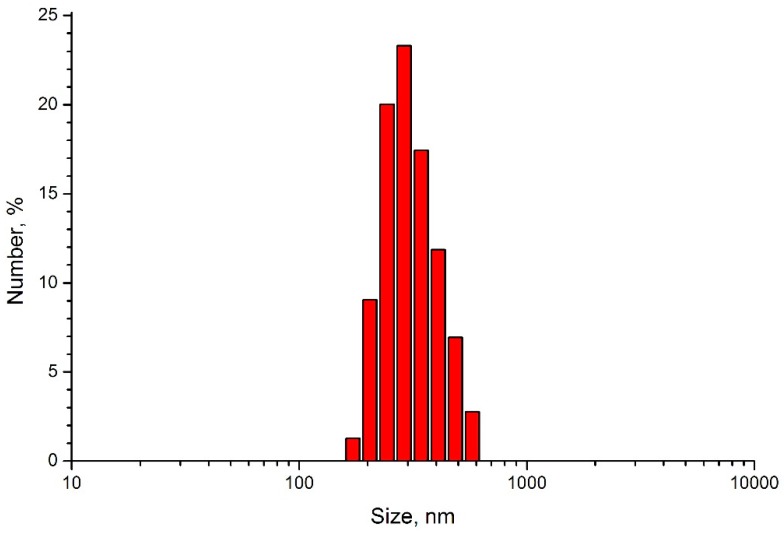
Size distribution of the DS–Dox NP.

**Figure 3:**
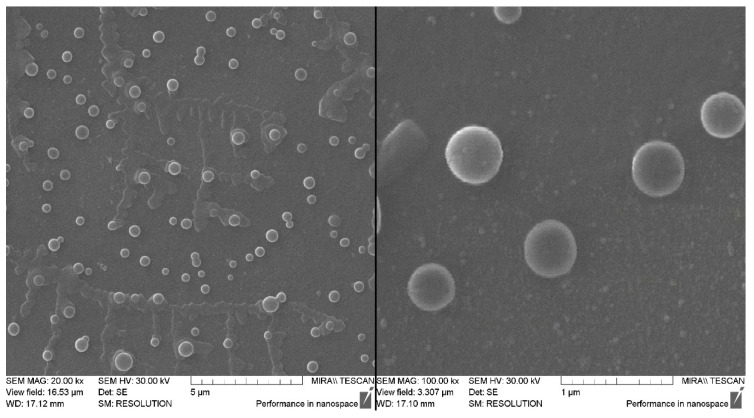
SEM imaging of DS–Dox NP.

We can see, the most homogeneous system with a high degree of drug loading was formed using a DS concentration of 15 mg/mL and a Dox concentration of 2.5 mg/mL (DS/Dox ratio = 3:1 m:m). These ratios were used for the synthesis of the DS–Dox NP, in further experiments. The particles synthesized under these conditions had the following characteristics—size 305 ± 58 nm; zeta potential –32.4 ± 2.8 mV; and a polydispersity index of 0.036. The concentration of doxorubicin in the colloid system, after dialysis, was equal to 0.85 mg/mL. The size distribution of the particles obtained by the chosen method is shown in Figure 2, and their SEM images are shown in Figure 3.

Thus, particles with a sufficient concentration of a chemotherapy agent, acceptable sizes, and homogeneity, were obtained. The literature describes examples of the formation of doxorubicin complexes with various anionic polymers, like polyacrylic acid [41] and γ-polyglutamic acid [42], due to the interaction between protonated primary amino groups of Dox and anionic groups of polymers. According to [43], a significant role in the formation of such stable systems is played by the intermolecular hydrogen bonds.

Doxorubicin, which is an anthracycline derivative, has a primary amino group, and shows basic properties. The Dox residue in salts is an organic cation, which is able to form ionic pairs with various anionic particles, including polyanions. Dextran sulfate is an anionic polymer constructed from α-D-glucopyranose residues linked by 1 → 6- and 1 → 3-glycosidic bonds [44]. The number of highly ionized sulfo-groups is about 2 per glycon, which makes possible the formation of DS–Dox complexes, due to the electrostatic interaction between cationic groups of Dox and anionic groups of DS. The presence of hydroxyl groups in the Dox, and the polymer molecules, makes the formation of hydrogen bonds possible. Therefore, both types of intermolecular interactions can take place in this case.

Dextran-sulphate and similar polysaccharides are quite water soluble. In a study by Yu et al. [45], data on a theoretical study of the molecular structure of sodium dextran sulfate in aqueous solutions, using the Merck Molecular Force Field method, were published. The authors found that various dextran sulfate oligomers in aqueous solutions were strongly solvated and existed in the form of spirals. The spiral conformation of the polysaccharide was due to the 1 → 6-glycosidic bonds, and the 1 → 3-glycosidic bonds, from peculiar branches on the helix. The spiral geometry was the most optimal, because it provided a minimal perturbation between the anionic groups in the adjacent monosaccharide blocks.

**Scheme 1:**
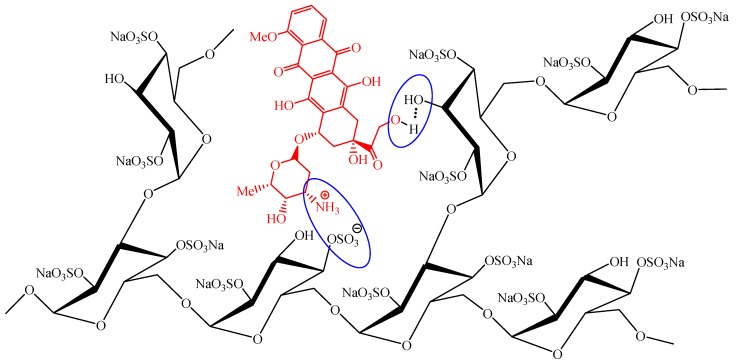
Interactions between the DS and Dox in nanoparticles (pH 6–7).

It is also known that dextran sulfate is soluble in polar organic solvents, like DMSO and DMF. However, in such solutions the geometry of the polymer molecules is different from the one described above. They look like a globule, since intramolecular hydrogen bonds between OH-groups, in different glycosidic units, are predominant. When a Dox solution in DMSO was added to an aqueous solution of dextran sulfate, the conformation of the macromolecule changed due to the enhancement of donor-acceptor interactions between different parts of the polymer molecule and the formation of closed cavities (“pockets”) containing polar (–SO_3_Na and –OH) groups. The change of the polysaccharide geometry was facilitated by the 1 → 6-glycosidic bond sites, around which free rotation was possible. With this mechanism of globule formation, the doxorubicin molecules got inside the DS globule, which ensured a high stability of the drug-polymer complex (Scheme 1).

It is noteworthy that when DMSO was removed during dialysis, the globular structure was preserved. This could be explained by the fact that the strength of intermolecular interactions inside the globule was greater than the strength of such interactions during solvation. An additional role in strengthening the bonds between the dextran chains, could have been played by the Dox molecules that acted as linkers. In addition, the globular structure itself could prevent the penetration of water molecules into the globule.

### 3.2. Release

**Figure 4:**
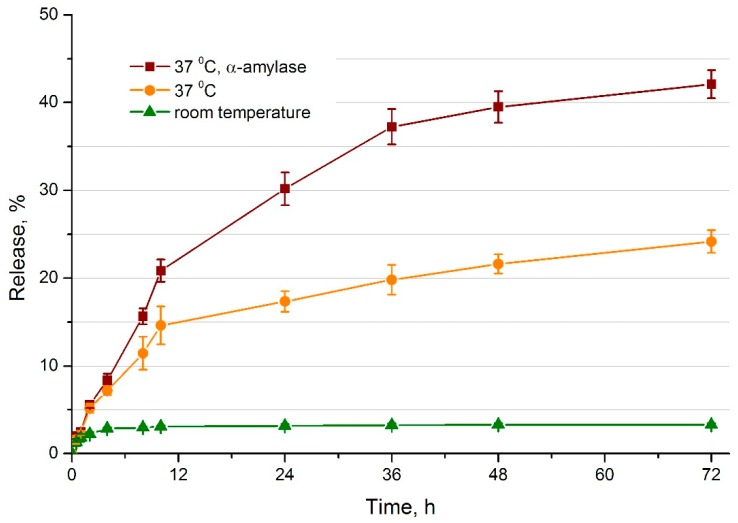
Dox release from the DS–Dox NP under various conditions.

The curves of the Dox release from the particles are shown in Figure 4.

At room temperature (control), the total release of Dox from particles was no more than 3.5%. This confirmed the stability of the synthesized complex.

Rising temperatures led to an increase in Dox release. At 37 °C, the maximum release was 24.2%. The release rate was maximum in the first 8 h, and then reached a plateau by 36 h. Increasing release with rising temperatures was expected and could be explained by an increase in the energy of the thermal vibrations of molecules, leading to a rupture of the hydrogen bonds between the hydroxyl groups of Dox and the polymer. This is consistent with the results obtained by Yousefpour et al. [22], who also observed an incomplete dissociation of similar complexes.

Of great interest was the release of Dox from the globules, in the presence of alpha-amylase. In this series, the cumulative release was 42.1%, which was almost 1.74 times higher than that in similar conditions without amylase. The most intense release was in the first 12 h, the plateau was reached by 48 h.

To study the effect of amylase on the nanoparticles, the dimensional characteristics of the globules in the presence of this enzyme and without it, were evaluated. In addition, the amount of free polymer after the treatment of particles with amylase, was studied. We expected that the amylase would reduce the size of the globules and increase the number of “free” DS molecules in the solution. However, the results of these experiments were contrary to what was expected. Treatment with amylase resulted in an increase in the average hydrodynamic particle diameter (up to 4.2 μm by 10 h of observation) and a two-fold decrease in the amount of free polymer. After 6 h, separation of the suspension was noted, and after 10 h, a thick precipitate was formed.

A possible explanation of the observed phenomenon might be the following. It is known that tumor-produced amylase belongs to a Saliva-type enzyme and breaks down 1 → 4 glycosidic bonds and the neighboring 1 → 6 bonds [46,47]. It could be assumed that, in this study, enzymatic hydrolysis of 1-6 or 1-3 glycosidic bonds had occurred in the polysaccharide molecule, at branch sites. Such destruction led to the opening of “pockets” and the appearance of long linear chains of DS on the surface of the globules. The latter induced a partial release of the accumulated Dox. Increase in the aggregation of particles could be caused by the electrostatic interaction between the sulfo- and hydroxyl groups of linear chains located on the surface of “neighboring” globules.

### 3.3. Cytotoxicity

**Figure 5:**
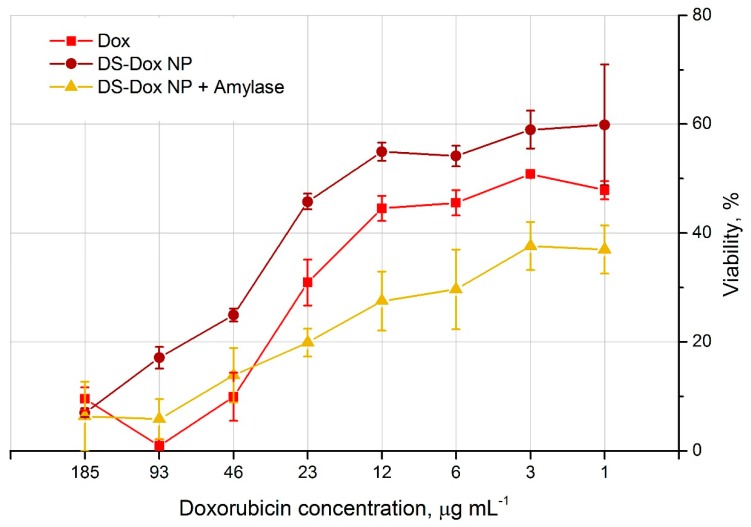
Viability of the L929 cells (MTT-test) under various conditions relative to the control (100%). Cell viability under the action of amylase equaled to 81 ± 3%.

The analysis of the viability of the L929 cells treated with the test substances, is presented in Figure 5.

Both, free Dox and the DS–Dox NP showed a significantly reduced cell viability, compared to the untreated cells. At concentrations of 1–10 µg/mL, there was no statistical difference between the toxicity of these drugs (*p* = 0.185–0.236), and in the concentration range of 20–185 µg/mL, the toxicity of DS–Dox NP was significantly less than doxorubicin (on average, by 10–15%, *р* = 0.034–0.495).

The cytotoxicity of the DS–Dox NP with amylase, was higher than without it, in all tested concentrations. In the low concentration range (1–12 μg/mL), it exceeded the cytotoxicity of the free Dox, and in the concentration range of 20–185 μg/mL, it did not significantly differ from it.

These statements were confirmed when the IC_50_ of the substances was calculated (Table 2).

For DS–Dox NP, the IC_50_ was higher than that for the free Dox, by 41%. Adding amylase to the incubation medium, reduced the IC_50_ for the DS–Dox NP. In this series of experiments, the IC_50_ was lower than that for, both, free Dox and DS–Dox NP, without amylase (2.6 and 4.5 times, respectively).

**Figure 6:**
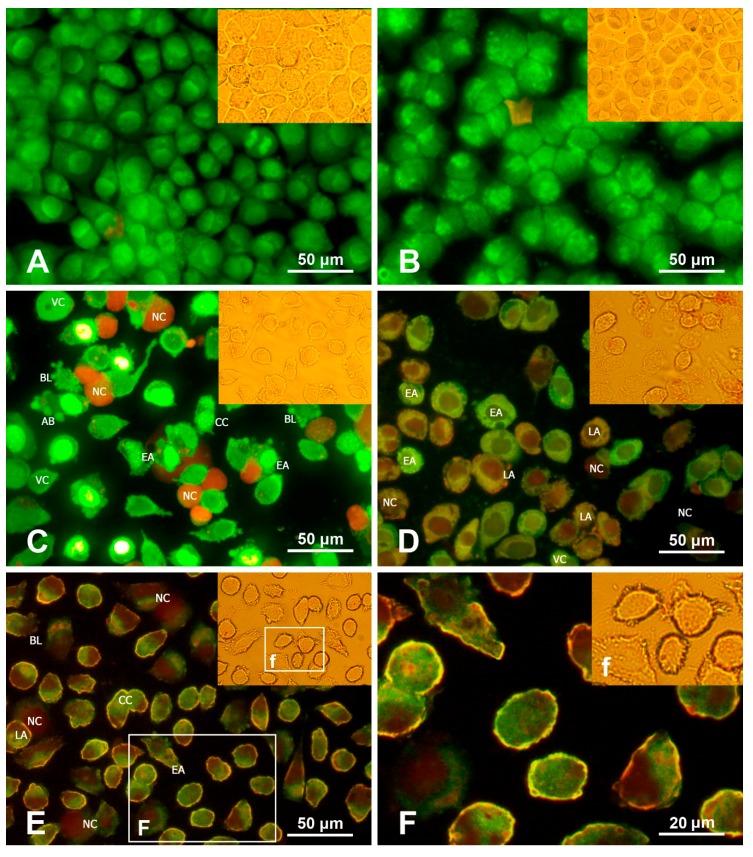
Fluorescent micrographs of the AO/PI-double-stained L929 cells (in the callouts—optic images). (**A**) Untreated cells, (**B**) cells treated with α-amylase 400 UA, (**C**) cells treated with doxorubicin 18.5 mkg/mL, (**D**) cells treated with DS–Dox NP 18.5 mkg/mL, (**E**,**F**) cells treated with DS–DoxNP and α-amylase 400 U/L, (**F**) fluorescent aureole phenomenon and (f) “relief”-like surface. Designation in the figures: VC—viable cells; EA—early apoptosis; LA—late apoptosis; NC—necrotic cells; CC—chromatin condensation; BL—blebbing of the cell membrane; and AB—apoptotic body.

The results of the evaluation of the morphology of cellular damage are presented in Figure 6.

Control cells with an intact membrane had a uniform green staining of the cytoplasm and the nucleus (Figure 6A).

Treatment with amylase at a dose of 400 U/L led to moderate cell damage; signs of early apoptosis were found (Figure 6B), but the large number of viable cells remained. The so-called “shrinkage” of cells was observed in these samples. The cells had lost their typical sprouts and had acquired a more rounded shape. At the same time, they did not lose contact with the well surface and the monolayer structure was remained.

In the series of cells treated with Dox, all signs of early and late apoptosis were observed—cytoplasm blebbing, chromatin condensation, and DNA fragmentation (Figure 6C). Single necrotic cells were also visualized and viable cells were practically absent. Optical microscopy also showed significant morphological changes—the monolayer was not dense, the integrity of cells was broken, the cells had a rounded form, and the cytoplasm “blebbing” and single apoptotic bodies were visible.

In the samples treated with DS–Dox NP, using optical and fluorescent microscopy, cells with both signs of apoptosis and necrosis were detected. It was noteworthy that the fluorescence intensity of the cells was lower than that in the samples with doxorubicin and the control samples. However, despite the lower intensity of fluorescence, viable, damaged, and necrotic cells can be differentiated from each other (Figure 6D).

Microscopy of samples treated with DS–Dox NP and amylase revealed interesting morphological features. Using optical microscopy, condensed (“relief”-like) surface of the monolayer was visualized. The contours of the cells were clear with a characteristic folding along the periphery (Figure 6E,F, callouts). In fluorescence microscopy, the luminescence intensity of the cells was found to be lower than that in other samples, however, bright staining of their contours was noted (Figure 6E,F). This phenomenon might be due to the precipitation of dextran–doxorubicin particles on the surface of the cell monolayer, as a polymer film, which formed the above-mentioned specific “relief” surface. This film most likely caused quenching of the cytoplasm glow and the appearance of a characteristic fluorescent aureole [39]. It was possible that this phenomenon had the same mechanism as that of DS–Dox NP condensation, under the action of amylase, which was observed in the experiments in vitro. However, in this case, there was no formation of the particle aggregates, but a polymer film appeared on the surface of the cell membrane, where glycocalyx was present. Glycocalyx is a carbohydrate polymer that showed a structural similarity to dextran and could also be depolymerized under the action of amylase. Probably, the formation of free sites for the binding of dextran caused the condensation of DS–Dox NP.

The results of a quantitative morphological assessment of viability correlated with the data obtained using the MTT test. The cytotoxicity of the free Dox was higher than its polymer-immobilized form (15% and 25% viable cell, respectively). The smallest number of viable cells (9%) was found in the series, where the cells were treated with DS–Dox NP and amylase. In this case, a possible mechanism of cytotoxicity might be a direct cell damage by the precipitation of DS–Dox NP and the formation of a “polymer film” on the cell surface. The layer of biopolymer could prevent the diffusion of gases and nutrients, and could disrupt the metabolic processes in the cells. In addition, it was possible that this “polymer film” prevented the release of doxorubicin from the cell and increased intracellular drug exposure, which enhanced the cytotoxic effect.

### 3.4. Coagulation

**Figure 7:**
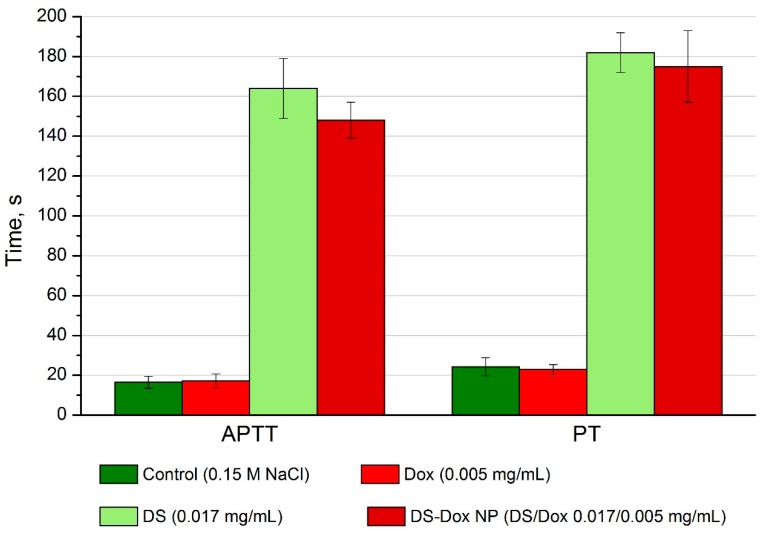
Changes of Activated Partial Thromboplastin Time (APTT) and Protrombin Time (PT) under the impact of DS–Dox NP and its components.

The effects of DS–Dox NP and its components on blood clotting are presented in Figure 7.

The DS–Dox NP prolonged, both, the APTT and PT (by 9.0 and 7.2 times, respectively, compared to the control). DS had demonstrated a similar effect. When added to the plasma, the APTT increased by 9.9 and PT by 7.5 times. Doxorubicin have shown no effect on the chronometric clotting tests. The mechanism of impact of the DS and its derivatives on blood clotting might be explained by the heparin-like effect of DS. The structural similarity of heparin and DS made possible the interaction of DS with the lysine residues in the Antitrombine-3 molecule [48]. The resulting complex inactivated the enzymatic coagulation factors and blocked both the external and the internal clotting pathway.

### 3.5. Antitumor Activity

The effectiveness of chemotherapy with the DS–Dox NP was higher in comparison with the free Dox (Table 3). In the animals treated with DS–Dox NP, a complete cure was recorded in 50% of the cases, whereas in the free Dox group it was 30%. The average life expectancy of the dead animals in the group treated with DS–Dox NP was 30% higher than the one in the group treated with the free Dox.

DS–Dox NP inhibited the hepatoma cell proliferation in a greater degree, compared with the traditional drug form. The volume of ascites in the group treated with DS–Dox NP was 21.5% lower, and the amount of tumor cells in the ascites decreased by 25.1%, compared with the group of free Dox (Table 4).

**Figure 8:**
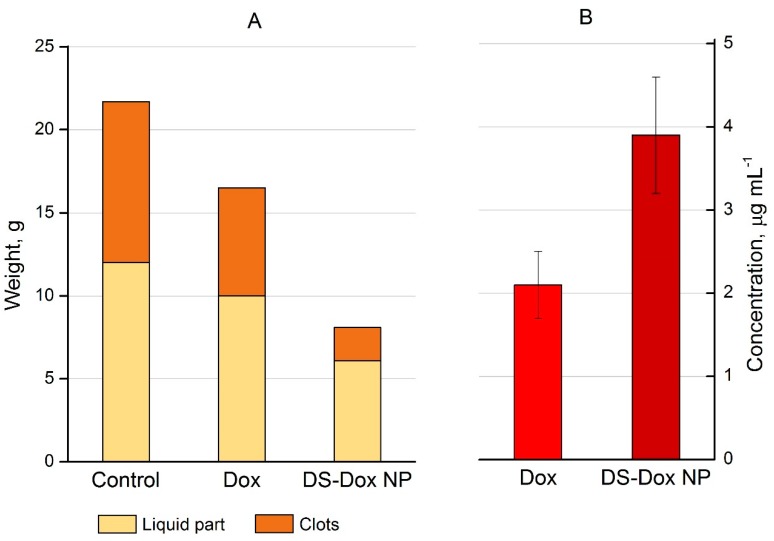
The total mass of ascites and the mass of fibrin clots in ascitic fluid (**A**) and the concentration of doxorubicin in the ascitic free fluid (**B**).

A possible reason for the increase in activity of the dextran-conjugated Dox was a change of its kinetics and distribution in the abdominal cavity, as well as its anticoagulant effect. The results of the evaluation of these indicators are presented in the Figure 8.

In the control group and in the group treated with free Dox, fibrin clots were detected in the abdominal cavity. In contrast, no thrombosis was observed in the ascitic fluid when DS–Dox NP were used. Due to the preservation of the liquid state, the drug was evenly distributed all over the abdominal cavity, and had contact with all of its structures. In addition, the DS–Dox NP were resorbed from the abdominal cavity, much slower than the free Dox. This could be a reason for the increasing drug exposure in the area of pathologic process. In the group treated with the DS–Dox NP, the drug concentration in the ascitic fluid was almost 2.5 times higher than that in the group treated with a free drug.

## 4. Conclusions

Increasing the effectiveness and reducing the toxicity of anticancer drugs is one of the main directions for improving chemotherapy. We have proposed a simple and convenient method for the synthesis of DS–Dox nanoparticles. This method provides a system suitable for creating a clinically applicable dosage form of the drug. When developing the composition, we assumed that it could be sensitive to amylase. Indeed, treatment with amylase resulted in an increase in the release of doxorubicin from the polymer complex; however, other phenomena, which might be relevant to the clinical use of the drug, were found. First, some unexpected effects of the amylase on the particles were found. In vitro, the particle size had not decreased but increase in the presence of this ferment and colloid system had coagulated. In a cell culture, a kind of polymer film was formed on the cell surface and this phenomenon had led to an increase in the particle cytotoxicity. Second, the anticoagulant effect of the synthesized particles on both the internal and external pathways of blood coagulation was revealed. It was known, that the anticoagulants prevented the formation of adhesions in the serous cavities, so we tested the synthesized particles on the model of ascitic tumor —Zajdel hepatoma in rats. DS–Dox NP had showed a higher efficiency in the treatment of this tumor, compared to free doxorubicin. The increase in efficiency was achieved not only by the anti-adhesive effect, but also by the creation of a higher concentration of the drug in the abdominal cavity. In this way, the synthesized particles could be promising medicine for the treatment of the intracavitary tumors, as well as the amylase-producing tumors.

## Figures and Tables

**Table 1:** Characteristics of the Dextrane Sulfate–Doxorubicin Nanoparticles (DS–Dox NP).

Concentration of DS in Water, mg/ml	Ratio DS:Dox, mg/mg	Size Particles, nm	Zeta-Potential, mV	Concentration of Dox in DS–Dox NP, mg/mL	PDI
Concentration of Dox in DMSO 1 mg/mL					
20	4:1	580 ± 61	−13.2 ± 1.7	0.45	0.011
15	3:1	389 ± 65	−22.4 ± 2.2	0.66	0.028
10	2:1	381 ± 67	−25.6 ± 3.0	0.48	0.031
5	1:1	240 ± 59	−27.8 ± 2.7	0.55	0.06
2.5	1:2	152 ± 38	−23.1 ± 1.8	0.44	0.063
Concentration of Dox in DMSO 2.5 mg/mL					
20	4:1	572 ± 104	−18.8 ± 2.1	0.64	0.033
15	3:1	305 ± 58	−32.4 ± 2.8	0.85	0.036
10	2:1	281 ± 70	−35.6 ± 1.7	0.55	0.062
5	1:1	145 ± 56	−31.2 ± 1.9	0.57	0.149
2.5	1:2	52 ± 21	−33 ± 2.7	0.45	0.163
Concentration of Dox in DMSO 4 mg/mL					
20	4:1	672 ± 112	−17.6 ± 1.5	0.29	0.028
15	3:1	451 ± 58	−29.2 ± 2.4	0.30	0.17
10	2:1	381 ± 49	−27.3 ± 2.8	0.26	0.17
5	1:1	245 ± 99	−24.3 ± 3.1	0.22	0.163
2.5	1:2	182 ± 67	−22.8 ± 2.9	0.16	0.136

**Table 2:** IC_50_ of the test substances according to the МТТ-test.

Parameter	Dox	DS–Dox NP	DS–DoxNP + Amylase (400 U/L)
IC_50_, μg/mL	2.85	4.82	1.08
95% confidence interval	0.449–6.62	2.59–7.70	0.413–1.93

**Table 3:** Antitumor effect of DS–Dox NP.

Groups	Mortality, %	Complete Cure, %	Life Expectancy of the Dead Animals, Days	Increase in Life Expectancy, %
Control (0.15 M sodium chloride)	100	0	7.4 ± 1.3	-
Dox (4 mg/kg)	70	30	11.5 ± 1.6	55.4
DS–Dox NP (DS/Dox 13.2/4 mg/kg)	50	50	16.2 ± 3.8	118.9

**Table 4:** Impact of the DS–Dox NP on the ascitic fluid.

Groups	The Frequency of Blood Clots in Ascitic Fluid, %	Ascite Volume, mL	The Number of Tumor Cells in Ascitic Fluid, Cells × 10^9^ /L	The Total Number of Cells in Ascitic Fluid, Cells × 10^10^ /L	Protein Concentration Cells in Ascitic Fluid g/L
Control (0.15 M sodium chloride)	40	26.7 ± 5.4	3.61 ± 0.51	1.32 ± 0.23	7.88 ± 1.22
Dox (4 mg/kg)	70	13.5 ± 4.3	2.23 ± 0.44	1.81 ± 0.30	6.54 ± 1.55
DS–Dox NP (DS/Dox 13.2/4 mg/kg)	10	10.6 ± 3.1	1.67 ± 0.42	0.65 ± 0.11	6.33 ± 0.89

## Supplementary Materials

The following are available online at https://www.mdpi.com/2073-4360/11/5/921/s1, Figure S1: Dependence of luminescence intensity on Dox concentration, Figure S2: Dose-response curve and IC_50_ calculation for amylase, Figure S3: Morphological assessment of the L929 cell changes, Table S1: Characteristics of DS–Dox NP complexes obtained in accordance with the method of Yousefpour et al., Table S2: Condition of synthesis and characteristics of DS–Dox NP.
