# Supplementary material for: Amylase-Sensitive Polymeric Nanoparticles Based on Dextran Sulfate and Doxorubicin with Anticoagulant Activity
Source: Polymers (Basel). 2019 May 25;11(5):921. doi: 10.3390/polym11050921 (PMC6571953; doi:10.3390/polym11050921)
Supplement: Supplementary file 1 [file polymers-11-00921-s001.pdf]

polymers

Supplementary Materials: Amylase-Sensitive Polymeric Nanoparticles Based on Dextran Sulfate and Doxorubicin with Anticoagulant Activity

Nikolay A. Pyataev, Pavel S. Petrov, Olga V. Minaeva, Mikhail N. Zharkov, Oleg A. Kulikov, Axeksandr V. Kokorev, Ekaterina P. Brodovskaya, Ivan A. Yurlov, Ilya V. Syusin, Andrey V. Zaborovsky and Larisa A. Balykova

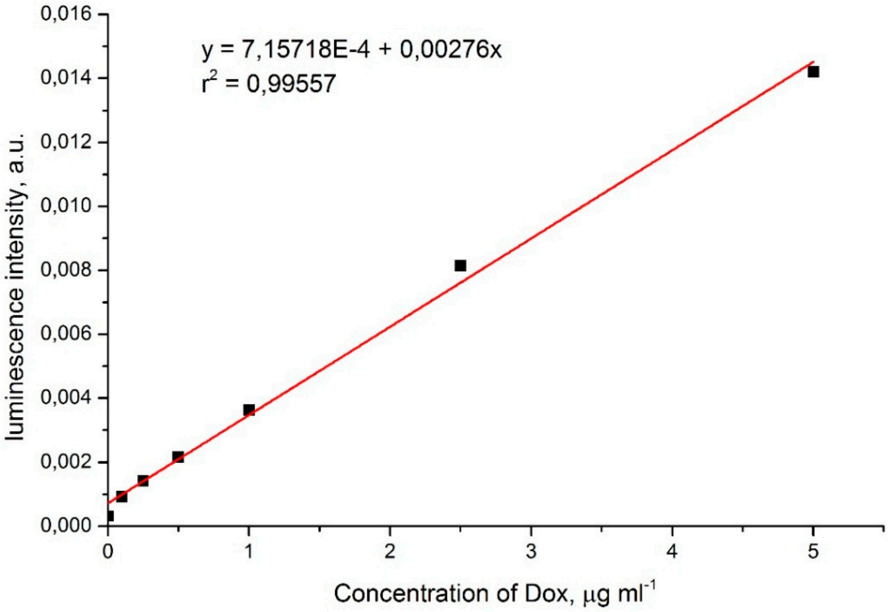

Figure S1. Dependence of luminescence intensity on Dox concentration. If the concentration of the substance was outside the linearity diapason of the calibration curve, the solution was diluted and the dilution factor was taken into account when calculating.

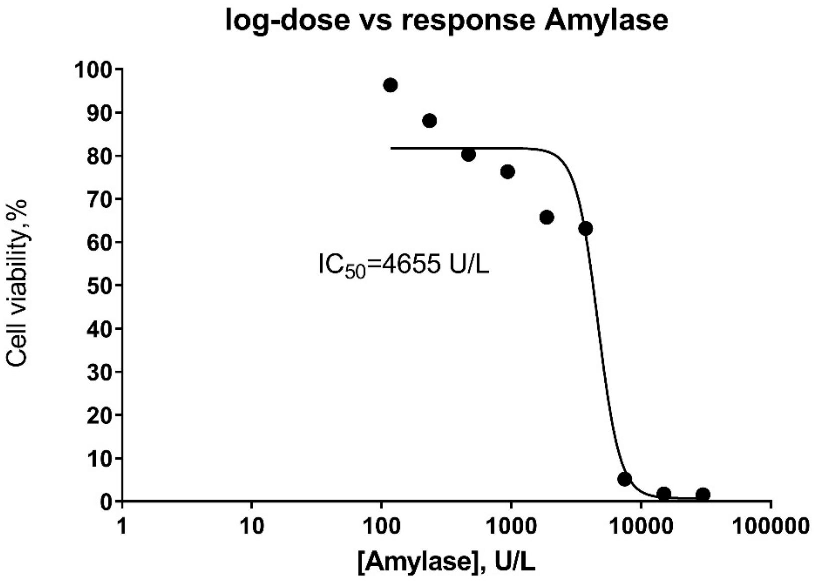

Figure S2. Dose-response curve and IC_{50} calculation for amylase.

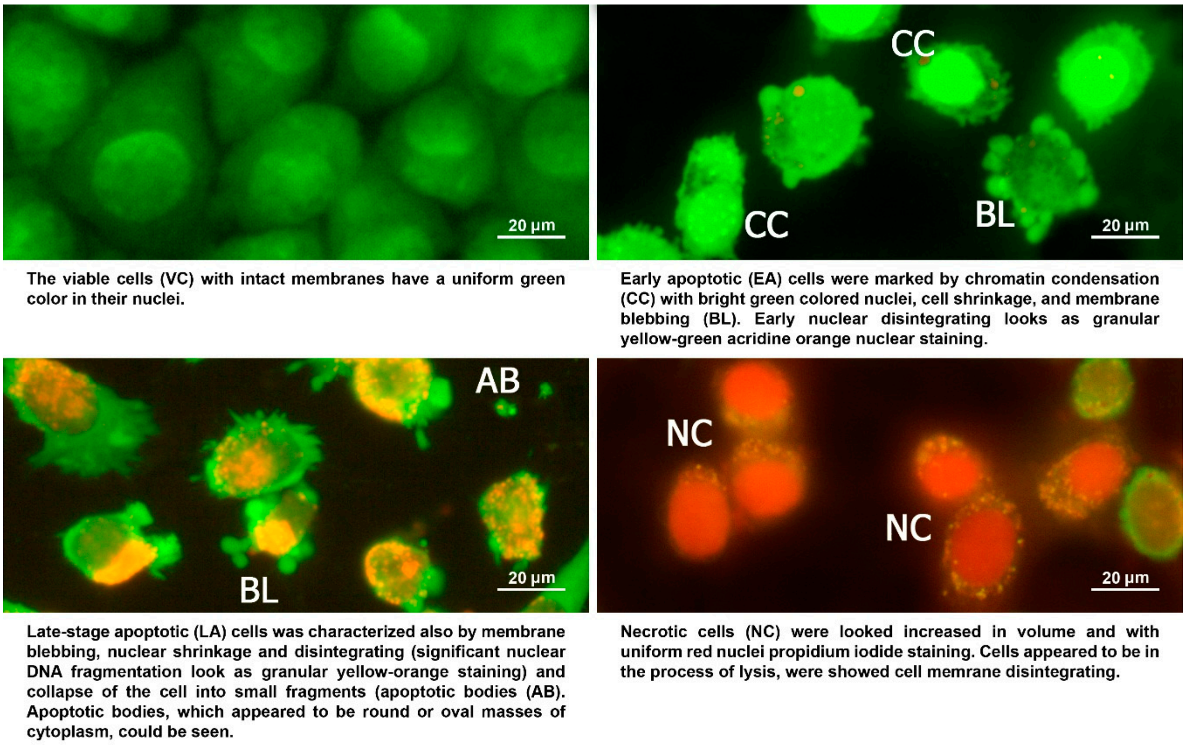

Figure S3. Morphological assessment of L929 cell changes (acridine orange and propidium iodide double staining).

Table S1. Characteristics of DS–Dox NP complexes obtained in accordance with the method of Yousefpour et al. [22]. .

Concentration of Dox, mg/ml	Concentration of DS, mg/ml	Ratio DS:Dox, mg/mg	Size particles, nm	Zeta potential, mV
0.1	0.04	4 : 10	480±38	-20.5±1.1
	0.06	6 : 10	290±23	-34.4±2.3
0.2	0.08	4 : 10	522±54	-24.1±1.7
	0.12	6 : 10	coagulation	coagulation
0.5	0.2	4 : 10	coagulation	coagulation
	0.3	6 : 10	coagulation	coagulation

Table S2. Condition of synthesis and characteristics of DS–Dox NP. .

Concentration of DS in water solution, mg/ml	Volume of DS water solution, ml	Concentration of Dox in DMSO, mg/ml	Volume of solution Dox in DMSO, ml	Volume of the reaction mixture, ml	Concentration of DS in reaction mixture, mg/ml	Concentration of Dox in reaction mixture, mg/ml	Ratio water: DMSO ml/ml	Ratio DS:Dox (m:m) in reaction mixture	Loading efficiency,%	Concentration of Dox in DS-Dox NP after dialysis, mg/ml	Ratio Dox :DS in DS-Dox NP, mg/mg
1	2	3	4	5	6	7	8	9	10	11	12
20	10	1	50	60	3.33	0.83	0.2	4	47	0.45	0.12
15	10	1	50	60	2.50	0.83	0.2	3	69	0.66	0.23
10	10	1	50	60	1.67	0.83	0.2	2	51	0.48	0.26
5	10	1	50	60	0.83	0.83	0.2	1	58	0.55	0.58
2.5	10	1	50	60	0.42	0.83	0.2	0.5	46	0.44	0.92
20	10	2.5	20	30	6.67	1.67	0.5	4	67	0.64	0.17
**15**	**10**	**2.5**	**20**	**30**	**5.00**	**1.67**	**0.5**	**3**	**89**	**0.85**	**0.30**
10	10	2.5	20	30	3.33	1.67	0.5	2	58	0.55	0.29
5	10	2.5	20	30	1.67	1.67	0.5	1	60	0.57	0.60
2.5	10	2.5	20	30	0.83	1.67	0.5	0.5	47	0.45	0.94
20	10	4	12.5	22.5	8.89	2.22	0.8	4	31	0.29	0.08
15	10	4	12.5	22.5	6.67	2.22	0.8	3	32	0.30	0.11
10	10	4	12.5	22.5	4.44	2.22	0.8	2	27	0.26	0.14
5	10	4	12.5	22.5	2.22	2.22	0.8	1	23	0.22	0.23
2.5	10	4	12.5	22.5	1.11	2.22	0.8	0.5	17	0.16	0.34

polymers

MDPI

© 2019 by the authors. Submitted for possible open access publication under the terms and conditions of the Creative Commons Attribution (CC BY) license (http://creativecommons.org/licenses/by/4.0/).
